# Dipeptidyl peptidase-4 inhibition and narrow-band ultraviolet-B light in psoriasis (DINUP): study protocol for a randomised controlled trial

**DOI:** 10.1186/s13063-016-1157-z

**Published:** 2016-01-15

**Authors:** Maeve Lynch, Tomás B. Ahern, Irene Timoney, Cheryl Sweeney, Genevieve Kelly, Rosalind Hughes, Anne-Marie Tobin, Donal O’Shea, Brian Kirby

**Affiliations:** Dermatology Department, St Vincent’s University Hospital, Elm Park, Dublin 4, Ireland; Endocrinology Department, St Vincent’s University Hospital, Elm Park, Dublin 4, Ireland; Dermatology Department, Adelaide and Meath Hospital Incorporating the National Children’s Hospital, Tallaght, Dublin 24, Ireland

**Keywords:** Psoriasis, Dipeptidyl peptidase-4 inhibition, Sitagliptin, Psoriasis area and severity index, Narrow-band ultraviolet-B phototherapy

## Abstract

**Background:**

Moderate to severe psoriasis is a systemic inflammatory disease associated with insulin resistance, obesity and type 2 diabetes (T2DM). Sitagliptin is a dipeptidyl peptidase-4 (DPP-4) inhibitor that improves glycaemia and has a marketing authorisation for the treatment of T2DM. Non-immunosuppressive therapies that are effective for psoriasis and its associated comorbidities would be a significant advance in the treatment of this chronic disease.

**Methods/Design:**

This is a single centre, 39-week, prospective, randomised, open label, clinical trial of oral sitagliptin (Januvia^®^) in psoriasis patients who are due to undergo a course of narrow-band ultraviolet-B (NB-UVB) phototherapy. We plan to enrol 120 participants and allocate participants on a random and 1:1 basis to receive sitagliptin 100 mg daily for 24 weeks combined with NB-UVB or NB-UVB monotherapy. Participants will be followed up for 12 weeks after sitagliptin therapy is discontinued. The primary endpoint is the change in Psoriasis Area and Severity Index (PASI) 24 weeks after treatment initiation. Secondary endpoints include cumulative NB-UVB dose, number of NB-UVB treatments required to clear psoriasis, proportions of participants who achieve PASI-50 (50 % reduction in PASI from baseline), PASI-75, PASI-90 and the proportion of participants who relapse in each group. We will also analyse changes in cardiovascular disease risk factors, serum cytokine and hormone levels and peripheral blood mononuclear expression of immune proteins at 24 and 36 weeks. A subgroup of participants will have skin biopsies taken and analysed for skin levels and expression of immune cells, receptors, hormones and immune proteins. The genetic or epigenetic profile that predicts best response to DPP-4 inhibitor therapy will be analysed. The safety endpoints include the rate and severity of adverse events.

**Discussion:**

This is the first randomised clinical trial assessing dipeptidyl peptidase-4 inhibition therapy in psoriasis. We hypothesise that sitagliptin therapy in combination with NB-UVB improves psoriasis severity compared to NB-UVB monotherapy.

**Trial registration:**

ClinicalTrials.gov Identifier NCT02347501 (Date of registration: 27 January 2015).

## Background

Psoriasis is a common immune-mediated skin disease affecting 1.3–2.2 % of the UK population [1] with chronic plaque psoriasis being the most common (90 %) form [2]. The majority of patients have mild psoriasis although around 20–30 % of patients have more severe involvement that warrants consideration of systemic therapy [3]. Treatment options for severe psoriasis include systemic immunosuppressant therapies such as methotrexate and fumaric acid esters which are effective but associated with potential toxicities. Biologic therapies have revolutionised the management of psoriasis but are expensive and are immunosuppressive. Psoriasis has a significant impact on quality of life and those patients with severe disease have an approximately 4-year reduction in life span [4]. Moderate to severe psoriasis is associated with smoking, alcohol excess, obesity and type 2 diabetes (T2DM) [5]. These comorbidities likely contribute to increased cardiovascular risk and premature mortality [6] as does the systemic inflammation associated with psoriasis [7]. An effective treatment for psoriasis that is non-immunosuppressive and treats comorbidities such as diabetes would, therefore, be very welcome.

Sitagliptin is a dipeptidyl peptidase-4 (DPP-4) inhibitor that has a marketing authorisation for T2DM. Sitagliptin has placebo-like tolerability and a good safety profile. Two case reports have shown that sitagliptin improved psoriasis severity [8, 9]. Dipeptidyl peptidase-4 is expressed on keratinocytes and its activity is upregulated in psoriasis [10, 11]. The main site of DPP-4 activity is CD26. CD26 is a marker of T cell activation and is a key molecule in the pathogenesis of autoimmune diseases [12]. Agents used to treat psoriasis commonly target the underlying inflammation [13, 14]. C-reactive protein (CRP) is a sensitive, systemic marker of inflammation [15]. In people with T2DM DPP-4 inhibitor therapy decreases CRP concentrations [16–22]. Serum CRP concentrations correlate with psoriasis severity and interventions that decrease the CRP concentration may decrease also psoriasis severity [23–25].

We have shown previously, in psoriasis patients without T2DM (both lean and obese), that homeostasis model assessment of insulin resistance (HOMA-IR) values correlate with those of the Psoriasis Area and Severity Index (PASI, a measure of psoriasis severity: *r* = 0.49, *p* < 0.001) [26].

Medications that improve insulin action may also decrease systemic inflammation and improve psoriasis [27]. This study aims to assess the efficacy and safety of sitagliptin, a DPP-4 inhibitor, in patients with psoriasis without diabetes undergoing narrow-band ultraviolet-B (NB-UVB) phototherapy. We hypothesise that sitagliptin therapy improves psoriasis severity.

## Methods/Design

### Study design and study organisation

This will be a single centre, 39-week, prospective, randomised, open label, clinical trial of oral sitagliptin tablets (Januvia^®^) in psoriasis patients who are due to undergo a course of NB-UVB phototherapy. We plan to enrol 120 research participants in total. Participants will be allocated at random to receive either (a) 24 weeks of sitagliptin (Januvia^®^, 100 mg daily, or 50 mg daily for participants with moderate kidney disease) with NB-UVB phototherapy; or (b) NB-UVB phototherapy without any additional treatment. The primary outcome measure is the change in the psoriasis area and severity index (∆PASI) during 24 weeks of treatment. This parameter, along with secondary outcome measures, will be compared between the two groups. This study has been approved by the St Vincent’s University Hospital Ethics and Medical Research Committee and by the Health Products Regulatory Authority of Ireland.

This study will be carried out in compliance with the study protocol and in accordance with the sponsor/contract research organisations’ standard operating procedures. These are designed to ensure adherence to Good Clinical Practice (GCP) guidelines, as described in the International Conference on Harmonisation of Technical Requirements for Registration of Pharmaceuticals for Human Use Harmonised Tripartite Guidelines for Good Clinical Practice, 1996.

### Trial registration

Ethical approval number: DPIP-2012-02

Date of approval: 19 June 2013

ClinicalTrials.gov Identifier: NCT02347501

EudraCT: 2012-005483-10

### Data handling

All data will be stored securely. Details of outcome measures and adverse events will be documented in hospital healthcare records, in individual research participant case report forms and in an encrypted electronic database.

The study investigators will adhere to hospital protocols pertaining to healthcare record use and storage. To protect the research participant’s identity, a unique identification code will be assigned by the investigator, or authorised designee, to each study participant and used in lieu of the participant’s name when the investigator reports adverse events and/or other study-related data are reported.

### Participants

Potentially eligible research participants will be identified through use of a patient database and through review of healthcare records in St Vincent’s University Hospital. Potentially eligible research participants will be recruited in one of two ways by one of the study investigators or by a suitably qualified designee. One of these two ways will be during a clinic visit and the other will be by mailing a letter of invitation.

Psoriasis patients attending this centre who have a PASI greater than 7, and who are due to undergo NB-UVB phototherapy, will be considered potentially eligible research participants and will be invited to attend for a screening visit. Informed consent will be obtained from every participant in the trial. The PASI will be measured at every visit by a senior clinician, or suitably qualified designee. The PASI assessor will be blinded to the participant’s randomisation group. After a 3-week period, where research participants will not receive the investigational medicinal product, those research participants who meet all of the inclusion criteria and do not fall under any of the exclusion criteria (Table 1) will be allocated randomly, after stratification by glycated haemoglobin (HbA1c) level (HbA1c <38 mmol/mol or ≥38 mmol/mol), body mass index level (BMI <30 kg/m^2^ or ≥30 kg/m^2^) and previous response to NB-UVB (achieved remission within 25 exposures during most recent course of NB-UVB or not), either to arm A or to arm B. Those who have not been exposed previously to NB-UVB will be placed in the ‘did not achieve remission within 25 exposures’ group.

**Table 1:** Inclusion and exclusion criteria

Inclusion criteria	Exclusion criteria
1. Patients who have a diagnosis of generalised chronic plaque and/or guttate psoriasis	1. Patients with photosensitive disorders
2. Male and female patients aged between 18 and 75 years inclusive	2. Patients with diabetes mellitus
3. Patients with a PASI of 7 or greater at screening or baseline despite use of topical therapies	3. Patients who are receiving medications that can cause photosensitivity
4. Patients who are due to undergo NB-UVB light therapy	4. Patient who are receiving GLP-1 analogue therapy
5. Patients who have not required systemic psoriasis therapy during the past 8 weeks	5. Patients who have conditions that could be made worse by phototherapy (cataracts, epilepsy, etc.)
6. Patients who are unlikely to require systemic therapy for the duration of clinical trial involvement	6. Patients with allergy or hypersensitivity to Januvia^®^
7. Patients who have a negative pregnancy test at screening (women of child-bearing potential only)	7. Patients with any of the following conditions: severe kidney disease (estimated glomerular filtration rate of less than 30 ml/min/1.73 m^2^) severe heart disease (left ventricular ejection fraction less than 35 %) severe liver disease (alanine aminotransferase greater than 150 IU/L) severe lung disease (forced expiratory volume in 1 second or a forced vital capacity that is known to be less than 50 % of that estimated for a person of that age and gender)
8. Patients who are willing to sign voluntarily a statement of informed consent to participate in the study	8. Patients who have received NB-UVB light recently (within 8 weeks)
	9. Patients who are receiving currently or have received systemic therapy for psoriasis recently (within 8 weeks)
	10. Patients who have any other contraindications to Januvia^®^ as stated in its Summary of Product Characteristics
	11. Female patients of child-bearing potential who are pregnant, breastfeeding, or unwilling to practice an acceptable barrier and/or hormonal method of contraception during participation in the study – abstinence will be permitted only if it is in keeping with a person’s lifestyle
	12. Patients with any clinically significant chronic disease that might, in the opinion of the investigator, interfere with the evaluations or preclude completion of the trial
	13. Patients with a current or recent (within the past 4 weeks) acute serious illness, acute psychiatric illness or severe uncontrolled/unstable illness
	14. Patients who have been randomised into this study previously
	15. Patients who are participating in another clinical trial concurrently
	16. Patients who are participating in another clinical trial during the 12 weeks prior to study entry (i.e. screening visit)

*GLP-1* glucagon-like peptide-1 analogue, *NB-UVB* narrow-band ultraviolet-B phototherapy, *PASI* psoriasis area and severity index

In order to achieve this we have prepared eight randomisation lists using a web-based random generator programme (http://www.randomization.com). For each participant the investigator, or authorised designee, will chose the appropriate list and will add the participant’s identifier to the list in chronological order. This list will be thereby used to determine the study treatment which the participant will receive. Random allocation will occur at visit 2 (baseline visit) once all screening procedures required at visit 1 (screening visit) have been completed, once it has been confirmed that the participant satisfies all inclusion and exclusion criteria and once the participant completes the 3-week run-in period. Identification numbers will be assigned chronologically in consecutive, ascending order.

### Study treatments

**Figure 1:**
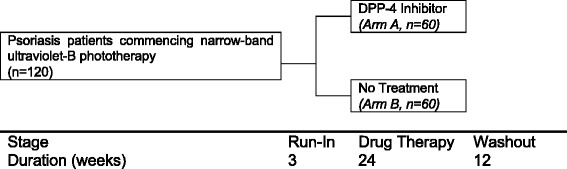
Depiction of the flow of research participants through each stage of the study: randomisation of research participants to one of two study arms, stages of no treatment and of treatment, and the medications that will be received by participants at each stage of the study

Research participants allocated to arm A will receive a 26-week supply of Januvia^®^ tablets (DPP-4 inhibitor) and will be instructed to ingest orally one 100 mg tablet once daily (or 50 mg once daily for participants with moderate kidney disease) for 24 weeks (Fig. 1). Research participants allocated to arm B will receive no treatment (aside from usual medications and phototherapy). Both the research participants and the investigators will be aware of the trial arm to which the research participant has been allocated randomly (open-label study).

All research participants will undergo NB-UVB phototherapy during the initial portion of study participation. This protocol involves whole-body NB-UVB using a Waldmann UV5001 cabinet incorporating 40 100-watt Philips TL-01 fluorescent lamps (emitting within the wavelength range of 311 nm to 313 nm) on Mondays, Wednesdays and Fridays. Doses of NB-UVB are adjusted according to the minimal erythema dose (MED) and according to erythemal response to therapy [28]. The MED is established by exposing eight 1.5 × 1.5 cm sites of unaffected skin of the upper back to NB-UVB at various doses (50, 70, 100, 140, 200, 280, 390, 550, 770 and 1080 mJ/cm^2^) from a bank of four TL-01 fluorescent tubes. The MED is defined as the dose that causes barely perceptible erythema 24 hours after irradiation. The first dose is 70 % of the MED and incremental increases are made at each visit to a maximum dose of 3833 mJ/cm^2^. Patients are treated until psoriasis clears or until they have received 40 exposures. All psoriasis patients wear protective UV goggles and the men wear genital protection. Irradiance is measured each month using an IL1400A radiometer and SEL240/UVB-1/TD detector head that is calibrated annually against a reference standard.

Research participants are prohibited from using systemic psoriasis therapy for the duration of their trial involvement. Any other medications that are considered necessary for the participant’s welfare and will not interfere with the study medication will be given at the discretion of the investigator.

### Safety

Comprehensive assessments of any apparent toxicity experienced by the research participant will be performed throughout the course of the study from the time of participant’s signature of informed consent. The safety of the investigational medicinal products will be assessed through the recording, reporting and analysing of baseline medical conditions, adverse events, vital signs and laboratory tests (full blood count, renal and liver blood tests). Information about all serious adverse events (SAEs) will be collected and recorded on the SAE Report Form. Each SAE must be reported by the investigator, or an authorised designee, to the sponsor within 24 hours of learning of its occurrence.

### Endpoints

Efficacy and safety measures will be performed according to a schedule of assessments (Table 2).

**Table 2:** Schedule of assessments

	Screening visit (week 3)	Baseline visit (day 0)	First treatment visit (week 3 ± 7 days)	Second treatment visit (week 6 ± 7 days)	Third treatment visit (week 12 ± 7 days)	Fourth treatment visit (week 24 ± 7 days)	First follow-up visit (week 30 ± 7 days))	End of study visit (week 36 ± 7 days)	Early withdrawal visit
Visit number	1	2	3	4	5	6	7	8	
Informed consent	X								
Inclusion/exclusion criteria	X								
Demographic data	X								
Medical history	X								
Quality of life assessments		X	X	X	X	X	X	X	X
Concomitant medication	X	X	X	X	X	X	X	X	X
Height		X							
Weight	X	X	X	X	X	X	X	X	X
Blood pressure and heart rate	X	X	X	X	X	X	X	X	X
PASI assessment	X	X	X	X	X	X	X	X	X
Blood samples^b^	X	X	X	X	X	X	X	X	X
Urinary pregnancy test (women of child-bearing potential only)	X	X						X	X
Adverse event assessment		X	X	X	X	X	X	X	X
IMP dispensing (arm A)		X							
IMP accountability return and compliance check			X	X	X	X			X^a^

^a^if applicable

^b^safety and efficacy variables

*IMP* Investigational Medicinal Product, *PASI* Psorasis Area and Severity Index

The primary efficacy endpoint is the change in the PASI (ΔPASI) after 24 weeks.

The secondary endpoints will include the:

1. ΔPASI after 36 weeks
2. Change in validated quality of life scores (Dermatology Life Quality Index (DLQI), EuroQol 5 item questionnaire (EQ-5D), Hospital anxiety and Depression Scale (HADS), and Stanford Health Assessment Questionnaire 8-Item Disability Scale (HAQ-8) after 24 and 36 weeks
3. NB-UVB light dosage and exposure number at 36 weeks
4. Proportion of patients who achieve a greater than 50 % reduction in PASI from baseline (PASI-50) by 36 weeks
5. Proportion of participants who achieve PASI-75 and PASI-90 by 36 weeks
6. Proportion of participants who relapse (PASI greater than 50 % of baseline value) by 36 weeks
7. Times taken to achieve PASI-50, PASI-75, PASI-90 and relapse
8. Changes in levels of cardiovascular disease risk factors (blood pressure, glycaemic measures lipid fractions, weight, etc.) after 24 and 36 weeks
9. Changes in serum concentrations of cytokines and hormones after 24 and 36 weeks
10. Changes in peripheral blood mononuclear cell expression of immune proteins (interleukin (IL)-6, tumour necrosis factor alpha (TNFα), IL-10, IL-17, interferon gamma, toll-like receptor 4, toll-like receptor 2, c-Jun N-terminal protein kinase 1, monocyte chemoattractant protein-1, etc.) after 24 and 36 weeks

The tertiary efficacy endpoints, which will be determined only in a subgroup of willing clinical trial participants, are the:

1. Changes in skin levels and expression of cells, hormones, receptors, enzymes and immune proteins after 24 weeks; and
2. Genetic, and/or epigenetic, profile that predicts best response to phototherapy and to DPP-4 inhibitor therapy

The safety endpoints will include:

1. Full blood count
2. Renal and liver blood tests
3. Skin reactions (erythema, pruritus, stinging and lesional blistering)
4. Reactivation of herpes simplex infection
5. Other adverse events

### Statistical methods and sample size

Demographic and baseline clinical data will be summarised using descriptive statistics according to treatment group. The primary and secondary efficacy variables and the safety variables will be summarised using descriptive statistics according to treatment group.

Data from research participants who are not allocated to either arm of the study *and* who do not receive a supply of investigational medicinal product will be excluded from statistical analyses.

The analysis of the data will be based on an intention-to-treat approach. *T* tests (or the appropriate alternative for non-parametric data), using two-sided tests, will be used to determine whether significant differences between the sets of data exist (SPSS version 20.0, IBM, Armonk, NY, USA). The independent samples *T* test will be used to assess for differences between the effects of the test product (Januvia^®^) compared to the effects of no additional treatment. Chi-square analyses will be used to test for significant differences in categorical variables between the sets of data obtained. Missing data will be dealt with using last observation carried forward.

Subgroup analyses will be performed on those research participants who:

1. Complete the visit 6 assessment
2. Have severe psoriasis
3. Have non-severe psoriasis
4. Are male
5. Are female
6. Are obese
7. Are older than 45 years

We plan to enrol 120 research participants in total.

Kleinpenning et al. have determined previously the effect of two different NB-UVB phototherapy regimens on the decrease in PASI 3 months after completion of a course of NB-UVB phototherapy [29]. Three months after cessation of NB-UVB light therapy the decrease in the PASI in those receiving an high-dose regimen was 5.93 ± 4.1 compared to baseline. This decrease in PASI was significantly less in the group receiving a low-dose regimen (4.14 ± 2.96, *p* < 0.05).

Based on these data, and assuming a 20 % dropout rate, we have calculated that we will require 60 research participants (in each randomisation arm) to detect a greater than 33 % difference in the ∆PASI with 80 % power and a 5 % significance level.

### Quality control of the study

A quality assurance audit may be conducted by the sponsor or its agent at any time during, or shortly after, the study. The investigator will permit an independent audit by an auditor mandated by the sponsor, after reasonable notice. The purpose of an audit is to confirm that the study is conducted as per protocol, Good Clinical Practice and applicable regulatory requirements, that the rights and well-being of the patients enrolled have been protected, and that the data relevant for the evaluation of the investigational medicinal product have been captured, processed and reported in compliance with the planned arrangements. The investigator will permit direct access to all study documents, drug accountability records, medical records and source data.

A future report will follow the Consolidated Standards of Reporting Trials (CONSORT) statement.

## Discussion

Psoriasis is characterised by keratinocyte hyperproliferation, by aberrant keratinocyte differentiation and by cutaneous inflammation [10].

The high concentration of DPP-4 expressed on keratinocytes and the fact that DPP-4 inhibition suppresses keratinocyte proliferation in vitro, and restores partially keratinocyte differentiation in vivo [30], support a potential role for DPP-4 inhibition therapy in the treatment of psoriasis. Two cases of DPP-4 inhibitor therapy improving psoriasis severity have been reported [8, 9]. One of these two cases was a woman in our department with severe psoriasis without diabetes who developed systemic B-cell lymphoma [9]. Treatment with methotrexate and acitretin were not tolerated or failed, and in the setting of the patient’s concomitant malignancy, sitagliptin was commenced in preference to an immunosuppressive therapy. After 8 weeks of oral sitagliptin treatment (100 mg once daily) the psoriasis body surface area (BSA) involvement decreased to less than 1 %.

DPP-4 inhibitors also prevent the degradation of insulin secretagogues such as glucagon-like peptide-1 (GLP-1), thereby ameliorating hyperglycaemia without causing hypoglycaemia [31]. Because of these effects, DPP-4 inhibitors are licensed for the treatment of T2DM. Other interventions that increase GLP-1 receptor activation, such as roux-en-Y gastric bypass surgery and GLP-1 analogue therapy, can also improve psoriasis severity [32–34].

We have previously reported a significant improvement in two patients with psoriasis and concomitant diabetes treated with the GLP-1 analogue liraglutide [33]. In a subsequent open study of seven patients with both psoriasis and type 2 diabetes we reported a significant reduction in psoriasis severity and a significant improvement in quality of life following treatment with liraglutide [35]. The median PASI decreased from 4.8 to 3.0 (*p* = 0.03) and median DLQI from 6.0 to 2.0 (*p* = 0.03). The improvement in patients treated with liraglutide was associated with an increase in circulating invariant natural killer T (iNKT) cells, innate T cells implicated in psoriasis pathogenesis, and a relative decrease in iNKT cell number in psoriatic plaques.

Another study evaluated seven patients treated with either exenatide or liraglutide for several months and found a significant reduction in PASI of 2.8 (from 12.0 ± 5.9 to 9.2 ± 6.4; *p* = 0.04) and a slight reduction in epidermal thickness (from 0.47 ± 0.12 to 0.40 ± 0.15 mm; *p* = 0.06). The decrease in PASI was associated with a decrease in Υδ-T cell number from 6.7 ± 4.5 to 2.7 ± 3.8 % (*p* = 0.05) and IL-17 expression (*p* > 0.05), both of which are involved in the pathogenesis of psoriasis [36]. These improvements in psoriasis are noted before weight loss occurs [33, 34, 36] and are not associated with improved glycaemic control [33–35].

A randomised clinical trial evaluated liraglutide in 20 obese glucose-tolerant patients with psoriasis [37]. After 8 weeks of treatment there was no significant difference in the change in PASI between the liraglutide group and the placebo group. There was a significant change in PASI from baseline in the liraglutide group and not in the placebo group. This was a small study (*n* = 20) and the study duration of 8 weeks was short.

Dipeptidyl peptidase-4 is expressed as CD26 on T cells. One hypothesis of sitagliptin’s potential effect in psoriasis treatment is that inhibition of DPP-4 may inhibit T cell activation and improve psoriasis. While there is evidence that sitagliptin decreases systemic inflammation, which may account for the reason it may be beneficial in psoriasis, there is no evidence to suggest that sitagliptin therapy is immunosuppressive. In one cross-over study of 36 patients with T2DM treated with sitagliptin or placebo, molecular markers of inflammation were altered significantly in the sitagliptin group [21]. Sitagliptin therapy (100 mg daily for 6 weeks) reduced serum concentrations of the inflammatory markers CRP, IL-6, IL-18 and reduced concentrations of the soluble cell adhesion proteins, intercellular adhesion molecule 1 and E-selectin (molecules involved in the development of atherosclerosis) in patients with T2DM. The change in CRP was correlated inversely with a rise in GLP-1 levels supporting a role for GLP-1 release, as well as with improved glucose-insulin homeostasis, in the improvement in systemic inflammatory and endothelial markers. A reduction in CRP has been demonstrated in other studies of patients with T2DM treated with sitagliptin [16–22] although no change in CRP has also been reported with sitagliptin therapy [38, 39]. One of the studies demonstrating the anti-inflammatory action of sitagliptin involved 22 obese patients with T2DM who were allocated at random to receive sitagliptin 100 mg daily or placebo for 12 weeks [22]. In this study peripheral blood mononuclear cell expression of the inflammatory molecules CD26 and TNFα decreased with sitagliptin therapy as did serum concentrations of inflammatory markers. Expression of CD26 decreased after a single dose of sitagliptin and the authors suggested that sitagliptin therapy, in addition to inhibiting the action of DPP-4, may inhibit synthesis of DPP-4.

Sitagliptin monotherapy has demonstrated improvements in measures of pancreatic β cell function (insulin secretion) as determined by the homeostasis model assessment-β cell function (HOMA-β) in the majority of studies evaluating both sitagliptin as monotherapy [40–44] or sitagliptin as an adjunctive therapy [45–49]. Improvements in insulin resistance have shown varied results. The majority of studies have shown improvements in insulin resistance when sitagliptin is used as an adjunctive treatment [45, 46, 48–50] rather than as monotherapy [41–44, 51]. One study of sitagliptin monotherapy has demonstrated that insulin resistance improved [40]. A meta-analysis of seven studies comparing sitagliptin to metformin found that sitagliptin was inferior to metformin in improving insulin resistance (*p* = 0.003) [52]. The majority of these studies measured insulin resistance using HOMA-IR, which correlates well with the euglycaemic clamp, which is the ‘gold standard’ for measuring insulin resistance [53]. Limitations associated with the use of HOMA-IR include lack of consensus regarding cut-off values and decreased reliability in lean patients with T2DM (due to lower β cell function and higher fasting glucose levels).

Sitagliptin treatment has not demonstrated immunosuppressive effects in healthy individuals. One study evaluated sitagliptin 100 mg daily compared to placebo for 28 days in healthy volunteers and measured immune function [54]. There was no effect on cytokines measured including the immunosuppressive cytokine, transforming growth factor β, on major lymphocyte subsets and on numbers of regulatory T cells suggesting that sitagliptin lacks immunomodulatory effects in healthy patients. A non-sustained increase in CD26 levels in T cells and increase in memory CD8^+^ T cells was observed. It is possible that immune effects may differ in patients with psoriasis, a chronic immune-mediated disease, and this trial should provide further insight into the effects of sitagliptin in psoriasis on immune function.

There are several other factors in support of sitagliptin therapy being non-immunosuppressive. Clinical trial data show no increase in the incidence of viral infections or malignancy with sitagliptin therapy [55]. Twenty patients with human immunodeficiency virus (HIV) who did not have T2DM were treated with sitagliptin 100 mg daily or placebo for 24 weeks and CD4+ T cell count and plasma HIV RNA levels were not affected by sitagliptin treatment [56]. There was a reduction in stromal derived factor-1α levels (required to protect T cells from HIV entry) in the sitagliptin group but this did not affect immune or virology status. These data support the lack of a non-immunosuppressive effect with sitagliptin.

In addition to improvements in hyperglycaemia, sitagliptin treatment has demonstrated improvements in other cardiovascular risk factors relevant to psoriasis patients, although results are inconsistent. Sitagliptin therapy has demonstrated improvements in blood pressure measurements in some studies of patients with T2DM [57–61] although other studies have shown no change in blood pressure with sitagliptin treatment [38, 62, 63]. The possible improvement in blood pressure may be related to GLP-1-related natriuresis and vasodilation. Similarly, improvements in lipid parameters have been reported in most [38, 50, 60, 64–66], but not all [39], studies of sitagliptin therapy.

Sitagliptin may also improve cardiac function. Pre-clinical studies have supported the cardioprotective effect of DPP-4 inhibition therapy [67–70], which may be mediated by stromal cell-derived factor-1 [70] associated with increased stem cell mobilisation. Two clinical trials evaluating other DPP-4 inhibitors, saxagliptin [71] and alogliptin [72], did not, however, find a reduction in cardiovascular events in patients with increased cardiovascular risk. The Trial to Evaluate Cardiovascular Outcomes after Treatment with Sitagliptin will be informative – this is an ongoing large prospective study evaluating the cardiovascular outcomes of patients with T2DM treated with sitagliptin [73].

Psoriasis is associated with multiple metabolic comorbidities including cardiovascular disease. Dipeptidyl peptidase-4 inhibitors, which reduce systemic inflammation and improve metabolic health, may improve psoriasis severity and may also provide an opportunity to treat, and prevent, major comorbidities. This study is the first prospective randomised clinical trial evaluating the potentially beneficial effects of DPP-4 inhibition in patients with psoriasis without diabetes.

## Trial status

Recruitment began in November 2013 and is ongoing.

## Abbreviations

∆PASI: the change in psoriasis area and severity index
BMI: body mass index
CD26: cluster of differentiation 26
CRP: C-reactive protein
DLQI: Dermatology Life Quality Index
DPP-4: dipeptidyl peptidase-4
EQ-5D: EuroQoL 5 item questionnaire
GLP-1: glucagon-like peptide-1
HADS: Hospital Anxiety and Depression Scale
HAQ-8: Stanford Health Assessment Questionnaire 8-Item Disability Scale
HbA1c: glycated haemoglobin
HIV: human immunodeficiency virus
HOMA-β: homeostasis model assessment-β cell function
HOMA-IR: homeostasis model assessment of insulin resistance
IL: interleukin
IMP: investigational medicinal product
iNKT cells: invariant natural killer T cells
MED: minimal erythema dose
NB-UVB: narrow-band ultraviolet-B phototherapy
PASI: Psoriasis Area and Severity Index
T2DM: type 2 diabetes mellitus
TNFα: tumour necrosis factor alpha
